# Higher Body Mass Index Is a Causal Risk Factor for Skin Infections: A Mendelian Randomisation Study Using UK Biobank and FinnGen

**DOI:** 10.1111/dom.70797

**Published:** 2026-04-20

**Authors:** Rhian Hopkins, Ethan de Villiers, Michael N. Weedon, Beverley M. Shields, John M. Dennis, Andrew P. McGovern, Harry D. Green

**Affiliations:** ^1^ Department of Clinical and Biomedical Sciences University of Exeter Medical School Exeter UK

**Keywords:** disease prevention, obesity care, observational study, real‐world evidence, weight management

## Abstract

**Aims:**

Infections remain a leading cause of mortality and morbidity globally. We aimed to evaluate the potential causal role of higher BMI on common bacterial, viral and fungal infections using Mendelian randomisation (MR).

**Material and Methods:**

In UK Biobank (*N* = 502 131, *N* = 230 542 with linked GP records), we tested observational associations and used one‐sample MR to test for a causal effect of BMI on common infections (skin infections, respiratory infections and urogenital infections) in primary care and hospital admissions. We additionally performed two‐sample MR using summary statistics from a BMI genome‐wide association study and infection outcomes from FinnGen (*N* = 500 348).

**Results:**

Higher BMI was observationally associated with all infection types. One‐sample MR demonstrated that higher BMI has a causal effect on skin infections in primary care (bacterial: Odds Ratio [OR] 1.37 [95% CI:1.24–1.53] per 5 kg/m2 increase in BMI, *p* < 0.001, fungal: 1.34 [95% CI:1.18–1.53], *p* < 0.001) and hospitalisation with skin infections (bacterial: OR 1.93 [95% CI:1.71–2.19] per 5 kg/m^2^ increase in BMI, *p* < 0.001, fungal: 2.81 [95% CI:1.58–4.97], *p* < 0.001). Two‐sample MR provided further evidence of a causal effect that is robust to pleiotropy. MR suggests that the estimated causal effect of higher BMI on some respiratory infections may be biased by pleiotropy.

**Conclusions:**

Mendelian randomisation provides strong evidence that higher BMI is a causal risk factor for bacterial and fungal skin infections. Weight loss interventions may help reduce the risk of both mild and severe bacterial and fungal skin infections and be targeted to those at highest risk.

## 1 Introduction

Infections are a major global health burden and remain a leading cause of mortality and morbidity [1, 2, 3]. Obesity is a global epidemic that affects over 1 in 8 people worldwide and continues to increase [4, 5, 6]. Higher BMI has been found to be associated with an increased risk of a range of common infections, including skin infections, respiratory infections, sepsis and urinary tract infections [7, 8, 9, 10]. This was further highlighted during the Covid‐19 pandemic when increased BMI was found to be associated with poor infection outcomes such as hospitalisation [11, 12, 13]. However, risk factor associations reported for BMI have been almost entirely observational. There is limited evidence evaluating the causality of this association and so the potential causal role of BMI in infections is uncertain.

Understanding what causes infections is necessary to devise interventions to reduce risk. Despite infections being a major public health issue, there are currently few guidelines on how to reduce the risk of infections. BMI is a potentially modifiable risk factor and so, if found to be causal, provides an intervention target for reducing infection risk. This is particularly important in high‐risk groups such as people with diabetes [14] and people with a history of recurrent or serious infection [15].

Causal inference methods such as Mendelian randomisation (MR) can be used to test for causal relationships using observational data. MR is an epidemiological method that uses genetic variation as an unconfounded proxy for the exposure (a person's genotype is randomly assigned at birth) to test for a causal relationship with an outcome [16, 17]. This method is becoming increasingly popular to overcome unmeasured confounding and reverse causality, major limitations in observational studies [16, 17, 18, 19].

There are few MR studies evaluating the causal role of modifiable risk factors for infections. One study assessing the association of BMI and infections has suggested a potential causal effect of BMI in hospitalisation for skin infections [20]. However, further research is needed to assess milder cases of infection treated in primary care, separate outcomes by infection aetiology and validate results in two‐sample MR. We therefore aimed to use an MR approach to test for a causal effect of higher BMI on common infections using large‐scale population‐based data.

## 2 Materials and Methods

### 2.1 Data

We used data from the UK Biobank and FinnGen, two of the largest datasets in the world combining genetic data and health information.

The UK Biobank is a large biomedical database containing health information and array‐based genotyping data on around 500 000 participants [21]. The UK Biobank participants were volunteers recruited aged 40–70 between 2006 and 2010 across the UK. The baseline data are linked to electronic health record data for hospital admissions, and in a subset (*n* = 230 542), primary care records.

FinnGen is a large‐scale genomics initiative that has analysed over 500 000 Finnish biobank samples and correlated genetic variation with health data to understand disease mechanisms and predispositions [22].

### 2.2 Outcomes

We studied a range of infection outcomes covering the three body systems most commonly associated with hospitalisation with infections: skin infections (bacterial skin infections, fungal skin infections), respiratory infections (bacterial pneumonia, influenza, non‐influenza viral respiratory tract infections) and urogenital infections (bacterial urinary tract infections, fungal genital infections). We also defined further subtypes of viral respiratory tract infections (lower respiratory tract infections, upper respiratory tract infections) and urinary tract infections (cystitis, pyelonephritis).

Using UK Biobank linked electronic health record datasets, for each of these infection types we defined two outcomes: infection treated in primary care and hospitalisation with infection. This allowed us to evaluate the potential impact of BMI on both mild and severe infection outcomes. We defined primary care infections as any Read code for the infection type in the primary care dataset. We defined hospitalisation as an International Classification of Diseases 10th Revision (ICD‐10) code for the infection type as any diagnosis in the Hospital Episodes Statistics (HES) dataset. The Read and ICD‐10 codes used to define each infection type are available in a Github repository: https://github.com/rhianhopkins/UKBiobank‐MRInfections. Individuals without a diagnosis of any of the studied infection types were used as controls in the respective analyses.

In FinnGen, we selected the closest matching outcomes to the ones we defined above in the UK Biobank. These were: L12_INFECT_SKIN (bacterial skin infections), AB1_DERMATOPHYTOSIS (fungal skin infection), J10_PNEUMOBACT (bacterial pneumonia), J10_INFLUENZA (influenza), J10_LOWERINF (lower respiratory tract infections), J10_UPPERINFEC (upper respiratory tract infections), N14_CYSTITIS (cystitis) and N14_PYELONEPHR (pyelonephritis). There was no closely matching outcome in FinnGen for fungal genital infections.

### 2.3 Exposure

We defined BMI using the values recorded in the UK Biobank baseline assessment data. Individuals with missing values for BMI were excluded from the analyses (*n* = 1409).

The genetic variants used for BMI were obtained from a 2015 genome‐wide association study (GWAS) of 339 224 individuals that reported 97 genome‐wide significant loci [23]. We excluded sex‐specific variants and those with potential pleiotropy or secondary signals within a locus, resulting in 72 variants used in our analysis. The 2015 GIANT consortium GWAS was chosen as it was conducted prior to the availability of UK Biobank genetic data and therefore did not include UK Biobank participants, minimising sample overlap between genotype–exposure and genotype–outcome datasets. Table S1 lists the genetic variants used and per‐SNP F‐statistics.

### 2.4 Statistical Methods

#### 2.4.1 Observational Associations

We tested for observational associations between BMI and infection outcomes using a logistic regression model adjusted for age and sex in the UK Biobank cohort. Effect sizes were reported as odds ratios per 5 kg/m^2^ BMI increase.

#### 2.4.2 Mendelian Randomisation Assumptions

Mendelian randomisation methods rely on three core assumptions: [24].

1. *Relevance*: the genetic instrument is associated with the exposure
2. *Independence*: the genetic instrument is not associated with confounders
3. *Exclusion restriction*: the genetic instrument influences the outcome only through the exposure

Additionally, an important assumption is gene–environment equivalence (the genetic instrument influences an environmental exposure equivalently to a proposed intervention that changes the population distribution of the exposure) [25].

#### 2.4.3 One‐Sample Mendelian Randomisation

We tested for a causal effect of BMI on infection using one‐sample MR in the UK Biobank cohort. We first combined genetic variants for BMI into a genetic risk score (GRS) using the published effect size for each SNP as weights. We excluded individuals from the subsequent analyses if we could not calculate a genetic risk score. In the first stage of the two‐stage least squares approach, the normalised GRS was regressed against BMI using a linear regression to derive genetically predicted exposure values. In the second stage, the genetically predicted exposure was regressed against the infection outcome in a logistic regression model, adjusted for age, sex and the residuals. Residuals are included to adjust for endogeneity in the exposure, reducing bias from confounding. Effect sizes were reported as odds ratios on the same scale as the observational associations.

We also stratified one‐sample MR by individuals with and without diabetes to assess for a potential causal role of BMI in people with diabetes who are at high risk of infections.

#### 2.4.4 Two‐Sample Mendelian Randomisation

To further evaluate the causal role of BMI in infections, we also used two‐sample Mendelian randomisation methods. This approach regressed the genotype‐exposure effect sizes against the genotype‐outcomes effect sizes for a set of SNPs associated with the exposure. We used inverse variance weighted (IVW) instrumental variable analysis and performed sensitivity analyses using additional methods that are more robust to potential violations of the standard MR assumptions (MR‐Egger, median IV, penalised median IV). MR Egger is robust to pleiotropy and assesses whether genetic variants have pleiotropic effects on the outcome that differ on average from zero (indicated by the intercept) [26, 27]. Median IV uses the median of the causal estimates for each genetic variant and allows up to 50% to be invalid instruments, and penalised median IV allows more precise causal estimates to contribute more weight to the analysis [27, 28]. We also used MR‐PRESSO to detect and correct for horizontal pleiotropy and outliers [29].

We obtained the effect sizes for the BMI genetic variants from the 2015 GWAS study described above (Table S1). To obtain genotype‐outcome associations for these SNPs, we used publicly available summary statistics from FinnGen [22] for each of the infection outcomes.

Statistical analysis was performed using R version 4.3.0, and STROBE‐MR reporting guidelines were followed [30]. R scripts used for analysis available at https://github.com/rhianhopkins/UKBiobank‐MRInfections.

## 3 Results

Of the 502 131 participants in the UK Biobank studied, 93 976 (18.7%) had a record of hospitalisation with infection. Of the 230 542 participants in the UK Biobank cohort who had linked GP records, 151 035 (65.5%) participants had a record of infection in primary care. The flow diagrams in Figures S1A and S1B show the numbers of each infection outcome and controls for each analysis.

Tables 1A and 1B describe the characteristics at UK Biobank assessment of those with and without infection and numbers of participants with each infection type. Those with an infection in primary care were more commonly female and more commonly had diabetes than those without an infection. Those with a hospitalisation with infection were on average older, had slightly higher BMI, had greater deprivation, more commonly had diabetes, and were more commonly smokers compared to those without. Baseline characteristics by infection type are reported in Tables S2A and S2B.

**TABLE 1A:** Baseline characteristics at UK Biobank assessment in individuals with linked GP data with and without a primary care infection.

	Infection	No infection
*n*	151 035	79 507
Sex (%)		
Male	64 536 (42.7)	39 965 (50.3)
Female	86 499 (57.3)	39 542 (49.7)
Age (mean (SD))	57.32 (8.02)	56.45 (8.11)
BMI (mean (SD))	27.75 (4.95)	27.11 (4.54)
Waist‐hip‐ratio (mean (SD))	0.87 (0.09)	0.87 (0.09)
Diagnosed diabetes (%)		
Yes	8835 (5.8)	3197 (4.0)
No	141 450 (93.7)	75 995 (95.6)
Unknown	750 (0.5)	315 (0.4)
Townsend deprivation index (mean (SD))	−1.34 (3.01)	−1.31 (3.08)
Smoking status (%)		
Current	16 044 (10.6)	8164 (10.3)
Previous	52 847 (35.0)	26 284 (33.1)
Never	81 288 (53.8)	44 701 (56.2)
Unknown	856 (0.6)	358 (0.5)
Infection (%)		
Bacterial skin infection	50 271 (33.3)	NA
Fungal skin infection	27 748 (18.4)	NA
Bacterial pneumonia	5716 (3.8)	NA
Influenza	13 594 (9.0)	NA
Non‐influenza viral respiratory tract infection	112 984 (74.8)	NA
Lower respiratory tract infection	76 158 (50.4)	NA
Upper respiratory tract infection	69 680 (46.1)	NA
Bacterial urinary tract infection	43 115 (28.5)	NA
Cystitis	42 008 (27.8)	NA
Pyelonephritis	2161 (1.4)	NA
Fungal genital infection	17 278 (11.4)	NA

**TABLE 1B:** Baseline characteristics at UK Biobank assessment in individuals with and without an infection hospitalisation.

	Infection	No infection
*n*	93 976	408 155
Sex (%)		
Male	47 002 (50.0)	181 972 (44.6)
Female	46 974 (50.0)	226 183 (55.4)
Age (mean (SD))	59.67 (7.69)	56.43 (8.06)
BMI (mean (SD))	28.62 (5.50)	27.16 (4.59)
Waist‐hip‐ratio (mean (SD))	0.90 (0.09)	0.87 (0.09)
Diagnosed diabetes (%)		
Yes	9784 (10.4)	16 602 (4.1)
No	83 494 (88.8)	389 636 (95.5)
Unknown	698 (0.8)	1917 (0.4)
Townsend deprivation index (mean (SD))	−0.82 (3.31)	−1.40 (3.03)
Smoking Status (%)		
Current	13 607 (14.5)	39 330 (9.6)
Previous	36 554 (38.9)	136 366 (33.4)
Never	42 990 (45.7)	230 336 (56.4)
Unknown	825 (0.9)	2123 (0.5)
Infection (%)		
Bacterial skin infection	24 433 (26.0)	NA
Fungal skin infection	1059 (1.1)	NA
Bacterial pneumonia	32 074 (34.1)	NA
Influenza	2005 (2.1)	NA
Non‐influenza viral respiratory tract infection	27 346 (29.1)	NA
Lower respiratory tract infection	22 668 (24.1)	NA
Upper respiratory tract infection	5612 (6.0)	NA
Bacterial urinary tract infection	37 900 (40.3)	NA
Cystitis	6218 (6.6)	NA
Pyelonephritits	3505 (3.7)	NA
Fungal genital infection	796 (0.8)	NA

The BMI genetic risk score was strongly associated with BMI (F‐statistic = 6243). All SNPs were strong instrumental variables (mean F‐statistics = 45, per‐SNP F‐statistics in Table S1A).

### 3.1 Mendelian Randomisation Demonstrates a Causal Effect of BMI on Skin Infections

**FIGURE 1:**
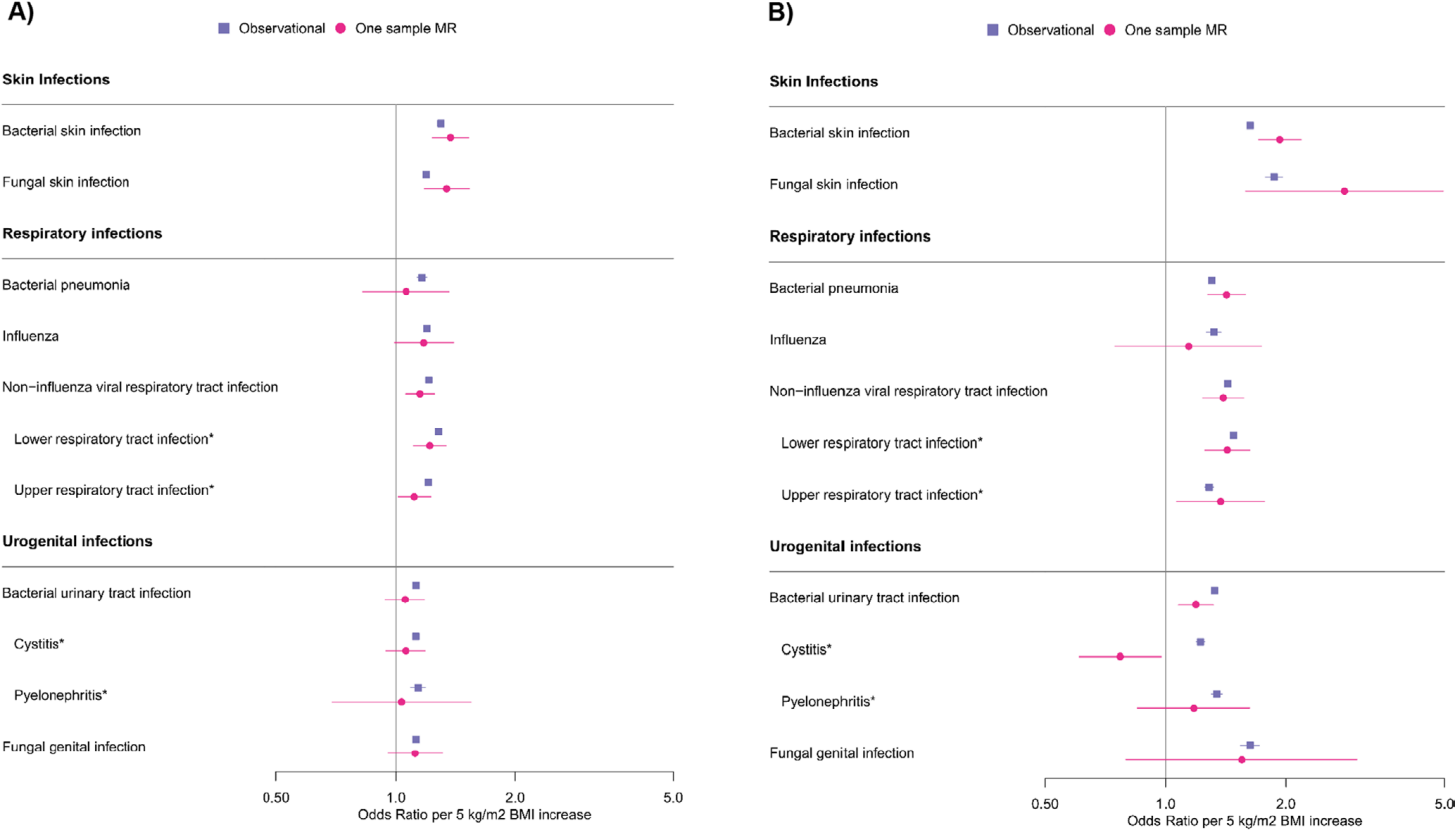
Forest plots of the observational (blue/purple squares) and one‐sample Mendelian randomisation estimates (pink circles) for the association of BMI and (A) infections in primary care and (B) hospitalisation with infection. Odds ratios and 95% confidence intervals are given per 5 kg/m^2^. *Lower respiratory tract infection and upper respiratory tract infection are subgroups of non‐influenza viral respiratory tract infection. Cystitis and pyelonephritis are subgroups of bacterial urinary tract infection.

Higher BMI was consistently observationally associated with skin infections, respiratory infections and urogenital infections in primary care and hospitalisation with these infections (Figure 1A,B).

**FIGURE 2:**
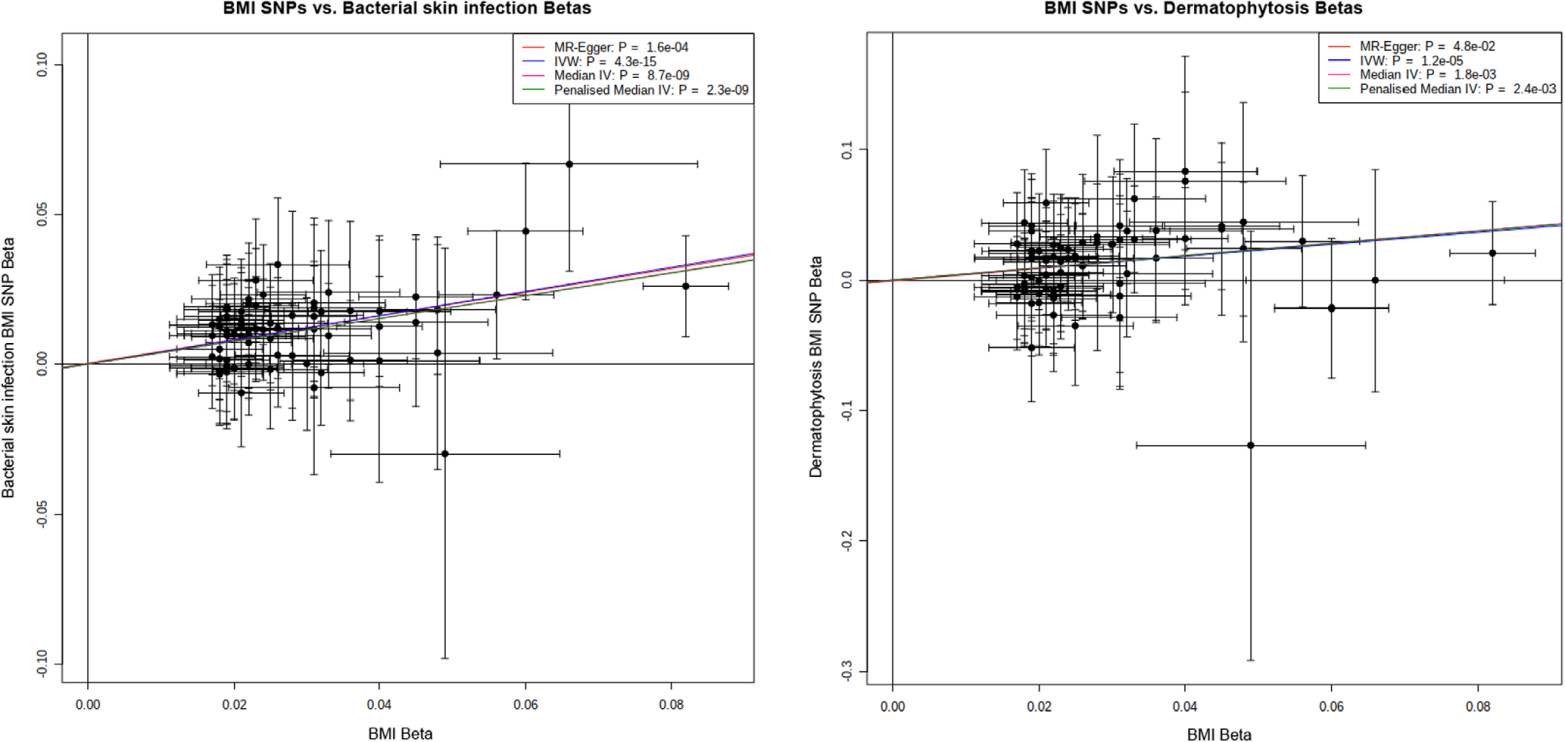
Two‐sample Mendelian randomisation results for BMI and FinnGen skin infection outcomes. BMI SNP effect sizes shown against effect sizes for (A) bacterial skin infections, and (B) dermatophytosis (fungal skin infection). IVW, MR‐Egger, median IV and penalised median IV tests used.

One‐sample Mendelian randomisation provides evidence that higher BMI has a causal effect on bacterial and fungal skin infections (Figure 1). A causal association with BMI was seen for skin infections both in primary care (bacterial skin infections: Odds Ratio [OR] 1.37 [95% CI: 1.24–1.53] per 5 kg/m^2^ increase in BMI, *p* < 0.001, fungal skin infections: OR 1.34 [95% CI: 1.18–1.53], *p* < 0.001, Figure 1B) and for hospitalisation with skin infections (bacterial skin infections: OR 1.93 [95% CI: 1.71–2.19] per 5 kg/m^2^ increase in BMI, *p* < 0.001, fungal skin infections: OR 2.81 [95% CI: 1.58–4.97], *p* < 0.001, Figure 1A). Two‐sample Mendelian randomisation provides further evidence of a causal role of higher BMI in bacterial skin infections and fungal skin infections, MR‐Egger sensitivity suggests no evidence of pleiotropy (Figure 2A,B), and MR‐PRESSO suggests no evidence of horizontal pleiotropy and detected no outliers (Table S3). Stratified one‐sample MR suggests a potential causal effect of higher BMI on bacterial and fungal infections in people with diabetes broadly consistent with those without diabetes (Tables S7 and S8).

One‐sample Mendelian randomisation suggests a potential causal effect of BMI for some respiratory tract infections (Figure 1). There was evidence that higher BMI may be causally associated with non‐influenza viral respiratory tract infections in primary care (OR 1.15 [95% CI 1.06–1.26], *p* = 0.001) and for hospitalisation (OR 1.39 [95% CI: 1.24–1.57], *p* < 0.001). Estimates for lower respiratory tract infections and upper respiratory tract infections were consistent with the overall association. There was also evidence of a potential causal association of higher BMI and hospitalisation with bacterial pneumonia (OR 1.42 [95% CI: 1.27–1.58], *p* < 0.001), but not bacterial pneumonia in primary care. However, two‐sample Mendelian randomisation MR‐Egger suggests bias by horizontal pleiotropy in the association of higher BMI and respiratory infections (Figure S2). MR‐PRESSO also suggests horizontal pleiotropy as well as outlier SNPs (Table S3).

One‐sample Mendelian randomisation did not show consistent evidence of a causal effect of BMI on urogenital infections. An association was seen for hospitalisation with bacterial urinary tract infection (OR 1.19 [95% CI: 1.07–1.32], *p* < 0.001); however, this was not consistent in primary care, or in the subgroup analyses of cystitis and pyelonephritis. Two‐sample Mendelian randomisation also suggests little evidence of a causal effect of BMI on urogenital infections (Figure S2).

Full numeric results and numbers included in the analysis of each infection type are given in Table S4 for the observational analysis, and Table S5 for one‐sample Mendelian randomisation. Table S6 provides full numerical results from two‐sample Mendelian randomisation.

## 4 Discussion

Using MR in large‐scale population‐based data, we demonstrate strong evidence of a causal role of increased BMI on bacterial and fungal infections. This causal effect was seen for milder cases of skin infection in primary care and hospitalisation with skin infections, and evidence was consistent in observational analyses and one‐ and two‐sample MR. The effect sizes identified are highly clinically relevant, with a doubling of risk for hospitalisation with skin infection for every 5 kg/m^2^ increase in BMI. Sensitivity analyses suggest this causal effect is robust to pleiotropy and outliers.

The causal estimate of higher BMI on some respiratory infections found in one‐sample MR analyses is suggested to be biased by horizontal pleiotropy in two‐sample MR sensitivity analyses. Genetic variants may be influencing respiratory infections through a pathway outside of BMI, which therefore violates the core MR assumptions. We also found little evidence of a causal effect of BMI on urogenital infections.

### 4.1 Interpretation/ Clinical Implications

Given the significant morbidity and mortality due to infections, the evidence we have found of a causal effect of BMI on bacterial and fungal skin infections provides a potential important target for intervention. As we found a causal association with infections in primary care and in hospital, this suggests that higher BMI could both cause increased risk of getting an infection and it being severe enough to be hospitalised. This evidence aligns with a previous study finding higher BMI was causally associated with increased risk of hospitalisation for skin infection [20]. Our study builds on this by providing evidence that the causal effect of higher BMI is consistent in both bacterial and fungal skin infections, and milder cases treated in primary care, and that the causal association is valid in two‐sample MR and robust to pleiotropy.

There are several plausible mechanisms, which may explain why higher BMI is causing infections. Intertrigo is a skin condition where increased skin folds result in areas of increased friction and moisture retention, making them susceptible to bacterial and fungal infections [31, 32]. Additionally, changes in blood flow within the skin due to obesity may impair the immune response to infections and lead to skin barrier impairment [32]. Skin conditions such as lymphoedema and venous insufficiency that are caused by obesity can also cause localised skin barrier breaches that can lead to infection [31, 32].

The robust evidence we provide demonstrating a causal role of BMI in skin infections highlights that weight management may be an important intervention target for skin infection prevention. Our MR estimates represent genetically increased BMI over a lifetime, and further research is needed to assess whether the same effect is also seen with short‐term pharmacological intervention. Weight loss interventions, including the increasing number of new medications such as the next generation incretin‐based agents [33], could potentially help reduce the risk of mild and severe bacterial and fungal skin infections, and this could merit investigation in randomised controlled trials. These weight loss interventions are expensive and therefore could be targeted to those most vulnerable to skin infections, for example people with diabetes [14] and people who have been admitted to hospital with a skin infection who are at high risk of being readmitted with another [15].

### 4.2 Strengths and Limitations

A key strength of this study is the use of two of the largest datasets combining genetic data and health‐related information that are available to study the causes of disease. The UK Biobank linked health record data allowed us to identify multiple common infection outcomes for a large cohort and the availability of systematically collected baseline data on BMI and genotyping data allowed us to test for causal associations using a one‐sample MR approach. The existence of a large GWAS study for BMI provided valuable sources of genetic information for the study exposures. The availability of summary statistics for genotype associations with a range of infection types from FinnGen allowed us to further validate these associations using two‐sample MR. The advantages of using MR to evaluate causal relationships are its robustness to confounding and reverse causation and can be used where conducting a randomised controlled trial is not possible. There are potential limitations to using data from the UK Biobank as the study recruited only individuals between the ages of 40–69 and there is a bias towards healthy individuals. The majority of UK Biobank participants are individuals of European ancestry and the numbers of individuals of other ancestries are too small to perform separate subgroup analyses in these groups. The summary statistics used in our two‐sample MR also came from studies of Europeans only, and so associations may not be generalisable to other ancestries.

We robustly defined infection outcomes both using the primary care data and hospital records, which allowed us to test both if the risk factors were causing increased infections and if they were causing more severe infections requiring hospitalisation. We also carefully defined and separated infection outcomes by whether they were likely bacterial, viral or fungal, which allowed us to test if the causal associations may differ by infection aetiology. In this study, we comprehensively evaluated a large range of infection outcomes covering the most common types of infection leading to hospitalisation. While we identified a significant causal effect of higher BMI on hospitalisation with fungal skin infections, the small number of events limited our statistical power to precisely estimate the magnitude of this effect. We were unable to use two‐sample MR to test for a causal association of BMI and fungal genital infections due to the lack of a close matching outcome in FinnGen; however, one‐sample MR suggested no evidence of a causal effect of higher BMI on this infection type. As with all studies involving health record data, misclassification of infection outcomes is possible and relies on correct coding in the records. In the UK, hospital coding is performed by professional clinical coders and primary care coding is usually performed by the clinician making the diagnosis. Using data from both sources mitigated against the impact of any systemic miscoding errors.

## 5 Conclusion

MR demonstrates a causal role of increased BMI on bacterial and fungal skin infections. Interventions, such as the newly available medications for weight loss, could help lower the risk of skin infections. This is particularly important in people at greatly increased risk of infections; for example, people with diabetes or a history of skin infection.

## Author Contributions

The study concept and design were conceived and developed by R.H., J.M.D., A.P.M. and H.D.G., R.H. and E.V. undertook the analysis, with support from H.D.G. All authors provided support for the interpretation of results, critically revised the manuscript and saw and approved the final article. H.D.G. attests that all listed authors meet authorship criteria, that no others meeting the criteria have been omitted. H.D.G. is responsible for the decision to submit for publication and is the guarantor of this work and, as such, had full access to all the data in the study and takes responsibility for the integrity of the data and the accuracy of the data analysis.

## Funding

J.M.D. is supported by a Wellcome Trust Early Career award (227070/Z/23/Z). This study was supported by the National Institute for Health and Care Research Exeter Biomedical Research Centre. The views expressed are those of the author(s) and not necessarily those of the NIHR or the Department of Health and Social Care.

## Disclosure

For the purpose of open access, the author has applied a Creative Commons Attribution (CC BY) licence to any Author Accepted Manuscript version arising from this submission.

## Ethics Statement

Ethics approval for the UK Biobank study was obtained from the North West Centre for Research Ethics Committee (11/NW/0382) [21].

## Consent

Written informed consent was obtained from all participants.

## Conflicts of Interest

A.P.M. received prior research funding from Eli Lilly and Company, Pfizer and AstraZeneca outside of the submitted work. All other authors declare no other relationships or activities that could appear to have influenced the submitted work.

## Supporting information


**Figure S1A.** Study flow diagram of individuals with linked GP data from UK Biobank and numbers of primary care infection outcomes.
**Figure S1B.** Study flow diagram of individuals from UK Biobank and numbers of hospitalisation with infection outcomes.
**Table S1.** Full list of BMI genetic variants used in Mendelian randomisation analysis.
**Table S2A.** Baseline characteristics by infection type (primary care) in individuals with GP data in UK Biobank.
**Table S2B.** Baseline characteristics by infection type (hospitalisation) in individuals in UK Biobank.
**Figure S2.** Two sample Mendelian randomisation results for BMI and FinnGen infection outcomes. BMI SNP effect sizes shown against effect sizes for (A) bacterial pneumonia, (B) influenza, (C) lower respiratory tract infections, (D) upper respiratory tract infections, (E) cystitis and (F) pyelonephritis. IVW, MR‐Egger, median IV and penalised median IV tests used.
**Table S3.** MR PRESSO sensitivity analysis results for pleiotropy and correction for outliers in the association of BMI and infections.
**Table S4.** Observational associations of BMI and infections in primary and infection hospitalisation.
**Table S5.** One sample Mendelian randomisation associations of BMI on infections in primary and infection hospitalisation.
**Table S6.** Two sample Mendelian randomisation results for BMI on infections. IVW, MR‐Egger, median IV and penalised median IV tests used.
**Table S7.** Observational associations of BMI and infections in primary and infection hospitalisation, stratified by individuals with/without diabetes.
**Table S8.** One sample Mendelian randomisation associations of BMI on infections in primary and infection hospitalisation, stratified by individuals with/without diabetes.

**Data S1.** dom70797‐sup‐0002‐supinfo.pdf.

## Data Availability

All individual‐level data used in this paper were obtained from the UK Biobank resource, and can be obtained from the UK Biobank at https://www.ukbiobank.ac.uk/enable‐your‐research/apply‐for‐access. Access to summary statistics from the FinnGen resource can be obtained at: https://www.finngen.fi/en/access_results/.
