# Supplementary material for: Higher Body Mass Index Is a Causal Risk Factor for Skin Infections: A Mendelian Randomisation Study Using UK Biobank and FinnGen
Source: Diabetes Obes Metab. 2026 Apr 20;28(7):5907–14. doi: 10.1111/dom.70797 (PMC13243997; doi:10.1111/dom.70797)
Supplement: Supplementary file 1 — Figure S1A. Study flow diagram of individuals with linked GP data from UK Biobank and numbers of primary care infection outcomes. Figure S1B. Study flow diagram of individuals from UK Biobank and numbers of hospitalisation with infection outcomes. Table S1. Full list of BMI genetic variants used in Mendelian randomisation analysis. Table S2A. Baseline characteristics by infection type (primary care) in individuals with GP data in UK Biobank. Table S2B. Baseline characteristics by infection type (hospitalisation) in individuals in UK Biobank. Figure S2. Two sample Mendelian randomisation results for BMI and FinnGen infection outcomes. BMI SNP effect sizes shown against effect sizes for (A) bacterial pneumonia, (B) influenza, (C) lower respiratory tract infections, (D) upper respiratory tract infections, (E) cystitis and (F) pyelonephritis. IVW, MR‐Egger, median IV and penalised median IV tests used. Table S3. MR PRESSO sensitivity analysis results for pleiotropy and correction for outliers in the association of BMI and infections. Table S4. Observational associations of BMI and infections in primary and infection hospitalisation. Table S5. One sample Mendelian randomisation associations of BMI on infections in primary and infection hospitalisation. Table S6. Two sample Mendelian randomisation results for BMI on infections. IVW, MR‐Egger, median IV and penalised median IV tests used. Table S7. Observational associations of BMI and infections in primary and infection hospitalisation, stratified by individuals with/without diabetes. Table S8. One sample Mendelian randomisation associations of BMI on infections in primary and infection hospitalisation, stratified by individuals with/without diabetes. [file DOM-28-5907-s002.pdf]

Supplementary tables/ figures

Supplementary figure 1A. Study flow diagram of individuals with linked GP data from UK Biobank and numbers of primary care infection outcomes.

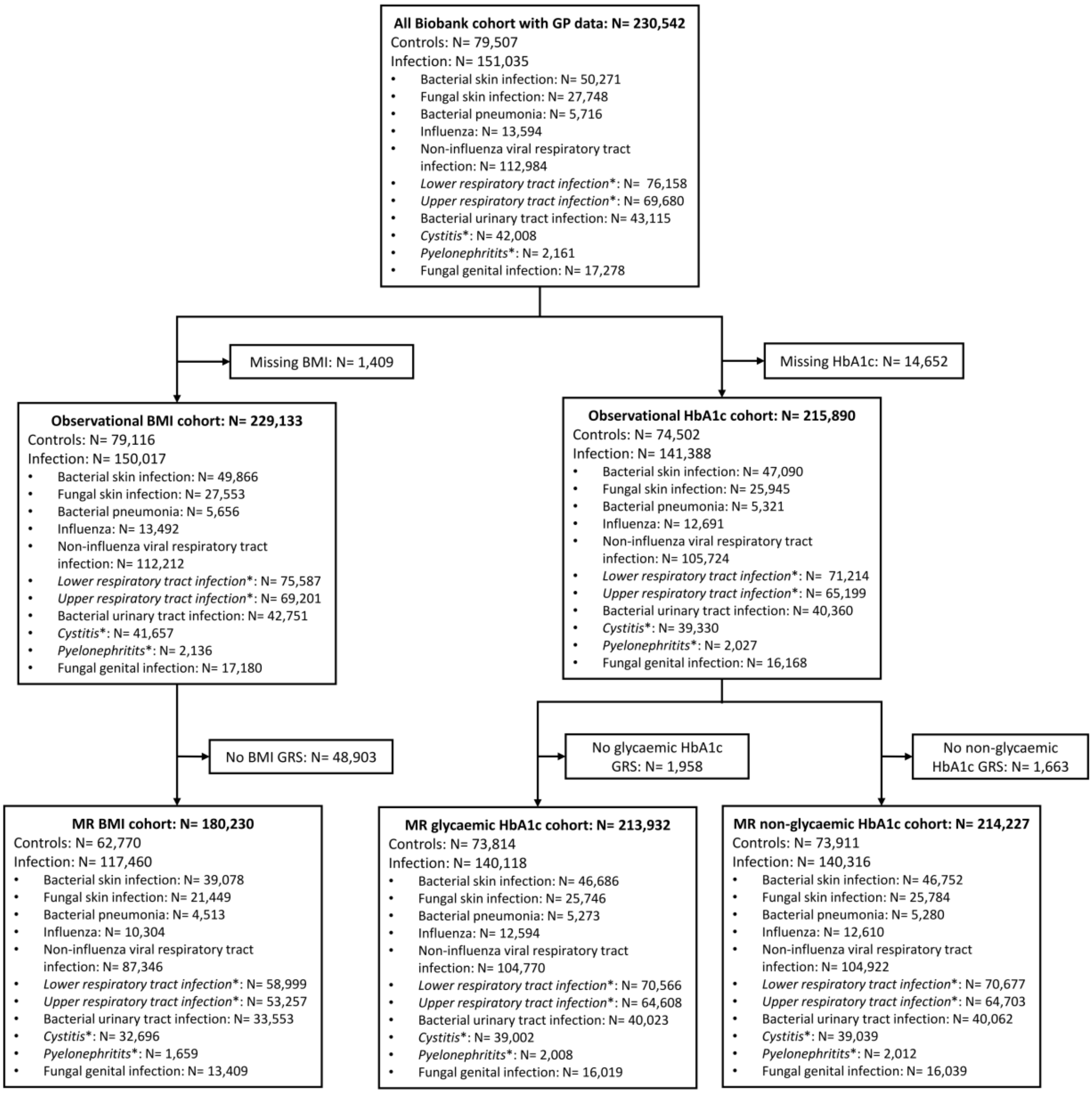

*Lower respiratory tract infection and upper respiratory tract infection are subgroups of non-influenza viral respiratory tract infection. Cystitis and pyelonephritis are subgroups of bacterial urinary tract infection.

Supplementary figure 1B. Study flow diagram of individuals from UK Biobank and numbers of hospitalisation with infection outcomes.

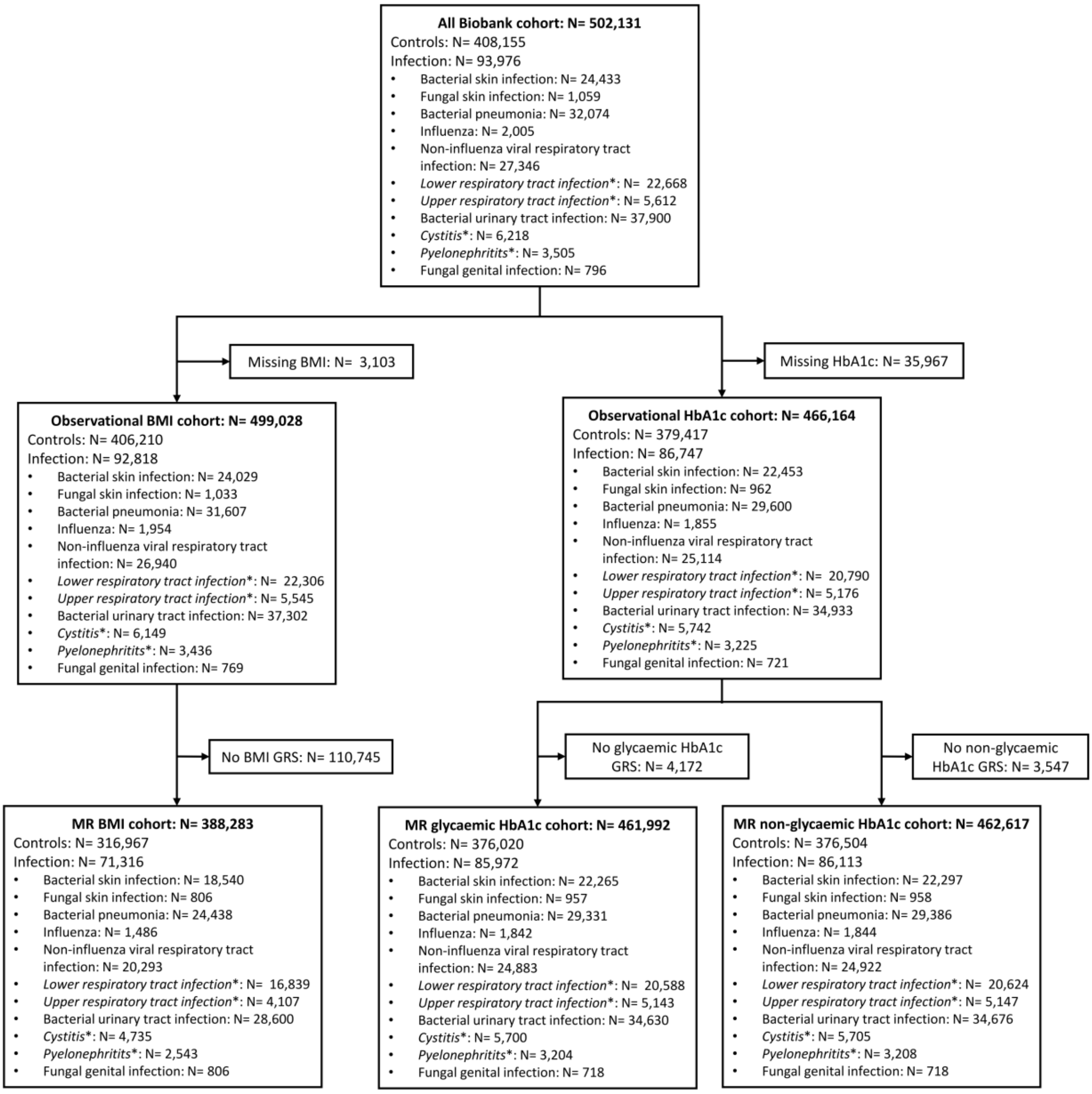

*Lower respiratory tract infection and upper respiratory tract infection are subgroups of non-influenza viral respiratory tract infection. Cystitis and pyelonephritis are subgroups of bacterial urinary tract infection.

Supplementary table 1. Full list of BMI genetic variants used in Mendelian randomisation analysis

SNP	Trait raising	Other allele	BetaXG	seBetaXG	F-statistic
rs10733682	A	G	0.017	0.003	32.1
rs11688816	G	A	0.017	0.003	32.1
rs1808579	C	T	0.017	0.003	32.1
rs6477694	C	T	0.017	0.003	32.1
rs11583200	C	T	0.018	0.003	36.0
rs12940622	G	A	0.018	0.003	36.0
rs1528435	T	C	0.018	0.003	36.0
rs29941	G	A	0.018	0.003	36.0
rs3736485	A	G	0.018	0.003	36.0
rs4740619	T	C	0.018	0.003	36.0
rs1000940	G	A	0.019	0.003	40.1
rs17724992	A	G	0.019	0.004	22.6
rs1928295	T	C	0.019	0.003	40.1
rs2033529	G	A	0.019	0.003	40.1
rs2033732	C	T	0.019	0.004	22.6
rs3849570	A	C	0.019	0.003	40.1
rs6804842	G	A	0.019	0.003	40.1
rs9400239	C	T	0.019	0.003	40.1
rs9925964	A	G	0.019	0.003	40.1
rs1167827	G	A	0.02	0.003	44.4
rs2176598	T	C	0.02	0.004	25.0
rs2365389	C	T	0.02	0.003	44.4
rs2820292	C	A	0.02	0.003	44.4
rs11126666	A	G	0.021	0.003	49.0
rs12401738	A	G	0.021	0.003	49.0
rs12885454	C	A	0.021	0.003	49.0
rs2650492	A	G	0.021	0.004	27.6
rs4256980	G	C	0.021	0.003	49.0
rs11165643	T	C	0.022	0.003	53.8
rs12286929	G	A	0.022	0.003	53.8
rs17405819	T	C	0.022	0.003	53.8
rs205262	G	A	0.022	0.004	30.3
rs7243357	T	G	0.022	0.004	30.3
rs7599312	G	A	0.022	0.003	53.8
rs10132280	C	A	0.023	0.003	58.8
rs1016287	T	C	0.023	0.003	58.8
rs657452	A	G	0.023	0.003	58.8
rs758747	T	C	0.023	0.004	33.1
rs12566985	G	A	0.024	0.003	64.0
rs7141420	T	C	0.024	0.003	64.0
rs10968576	G	A	0.025	0.003	69.4
rs17094222	C	T	0.025	0.004	39.1

rs2121279	T	C	0.025	0.004	39.1
rs2075650	A	G	0.026	0.005	27.0
rs2112347	T	G	0.026	0.003	75.1
rs3817334	T	C	0.026	0.003	75.1
rs13191362	A	G	0.028	0.005	31.4
rs3810291	A	G	0.028	0.004	49.0
rs13078960	G	T	0.03	0.004	56.3
rs10182181	G	A	0.031	0.003	106.8
rs11057405	G	A	0.031	0.006	26.7
rs11191560	C	T	0.031	0.005	38.4
rs16951275	T	C	0.031	0.004	60.1
rs17001654	G	C	0.031	0.005	38.4
rs2245368	C	T	0.032	0.006	28.4
rs7138803	A	G	0.032	0.003	113.8
rs12429545	A	G	0.033	0.005	43.6
rs3101336	C	T	0.033	0.003	121.0
rs11727676	T	C	0.036	0.006	36.0
rs2287019	C	T	0.036	0.004	81.0
rs10938397	G	A	0.04	0.003	177.8
rs12446632	G	A	0.04	0.005	64.0
rs7899106	G	A	0.04	0.007	32.7
rs1516725	C	T	0.045	0.005	81.0
rs2207139	G	A	0.045	0.004	126.6
rs16851483	T	G	0.048	0.008	36.0
rs543874	G	A	0.048	0.004	144.0
rs11847697	T	C	0.049	0.008	37.5
rs6567160	C	T	0.056	0.004	196.0
rs13021737	G	A	0.06	0.004	225.0
rs17024393	C	T	0.066	0.009	53.8
rs1558902	A	T	0.082	0.003	747.1

Supplementary table 2A. Baseline characteristics by infection type (primary care) in individuals with GP data in UK Biobank.

	No Infection	Bacterial skin infection	Fungal skin infection	Bacterial pneumonia	Influenza	Other viral respiratory infection	Lower respiratory infection	Upper respiratory infection	Bacterial urinary tract infection	Cystitis	Pyelonephritis	Fungal genital infection
n	79507	50271	27748	5716	13594	112984	76158	69680	43115	42008	2161	17278
**Sex (%)**												
Male	39965 (50.3)	23158 (46.1)	15088 (54.4)	2960 (51.8)	5643 (41.5)	47695 (42.2)	32793 (43.1)	26934 (38.7)	9434 (21.9)	9096 (21.7)	430 (19.9)	3732 (21.6)
Female	39542 (49.7)	27113 (53.9)	12660 (45.6)	2756 (48.2)	7951 (58.5)	65289 (57.8)	43365 (56.9)	42746 (61.3)	33681 (78.1)	32912 (78.3)	1731 (80.1)	13546 (78.4)
**Age (mean (SD))**	56.45 (8.11)	57.46 (8.01)	57.73 (7.89)	58.93 (7.69)	56.61 (7.92)	57.42 (8.01)	58.00 (7.89)	56.95 (8.05)	57.93 (7.91)	57.94 (7.90)	57.18 (8.06)	55.57 (8.14)
**BMI (mean (SD))**	27.11 (4.54)	28.37 (5.37)	27.95 (4.89)	27.85 (5.22)	27.90 (5.01)	27.94 (5.00)	28.25 (5.13)	27.90 (5.01)	27.50 (5.10)	27.49 (5.10)	27.56 (5.32)	27.43 (5.16)
**Waist-hip-ratio (mean (SD))**	0.87 (0.09)	0.88 (0.09)	0.89 (0.09)	0.89 (0.09)	0.87 (0.09)	0.87 (0.09)	0.88 (0.09)	0.87 (0.09)	0.85 (0.09)	0.85 (0.09)	0.85 (0.09)	0.84 (0.09)
**Diagnosed diabetes (%)**												
Yes	3197 (4.0)	3747 (7.5)	1808 (6.5)	485 (8.5)	809 (6.0)	6887 (6.1)	5240 (6.9)	4089 (5.9)	2593 (6.0)	2495 (5.9)	182 (8.4)	1240 (7.2)
No	75995 (95.6)	46267 (92.0)	25790 (92.9)	5203 (91.0)	12704 (93.5)	105502 (93.4)	70512 (92.6)	65197 (93.6)	40306 (93.5)	39305 (93.6)	1963 (90.8)	15961 (92.4)
Unknown	315 (0.4)	257 (0.5)	150 (0.6)	28 (0.5)	81 (0.5)	595 (0.5)	406 (0.5)	394 (0.5)	216 (0.5)	208 (0.5)	16 (0.8)	77 (0.4)
**Townsend deprivation index (mean (SD))**	-1.31 (3.08)	-1.29 (3.03)	-1.47 (2.96)	-1.14 (3.19)	-1.23 (3.06)	-1.31 (3.01)	-1.25 (3.04)	-1.32 (3.00)	-1.36 (2.99)	-1.37 (2.99)	-1.15 (3.08)	-1.41 (2.95)
**Smoking status (%)**												
Current	8164 (10.3)	6127 (12.2)	2687 (9.7)	798 (14.0)	1512 (11.1)	12236 (10.8)	9167 (12.0)	6803 (9.8)	4145 (9.6)	4002 (9.5)	254 (11.8)	1629 (9.4)
Previous	26284 (33.1)	18046 (35.9)	10083 (36.3)	2223 (38.9)	4651 (34.2)	39913 (35.3)	27753 (36.4)	24036 (34.5)	14828 (34.4)	14407 (34.3)	763 (35.3)	5554 (32.1)

Never	44701 (56.2)	25808 (51.3)	14800 (53.3)	2660 (46.5)	7343 (54.0)	60151 (53.2)	38770 (50.9)	38408 (55.1)	23880 (55.4)	23342 (55.6)	1128 (52.2)	9993 (57.8)
Unknown	358 (0.5)	290 (0.6)	178 (0.6)	35 (0.6)	88 (0.6)	684 (0.6)	468 (0.6)	433 (0.6)	262 (0.6)	257 (0.6)	16 (0.7)	102 (0.6)

Supplementary table 2B. Baseline characteristics by infection type (hospitalisation) in individuals in UK Biobank.

	No Infection	Bacterial skin infection	Fungal skin infection	Bacterial pneumonia	Influenza	Other viral respiratory infection	Lower respiratory infection	Upper respiratory infection	Bacterial urinary tract infection	Cystitis	Pyelonephritis	Fungal genital infection
n	408155	24433	1059	32074	2005	27346	22668	5612	37900	6218	3505	796
**Sex (%)**												
Male	181972 (44.6)	13366 (54.7)	625 (59.0)	18077 (56.4)	961 (47.9)	13874 (50.7)	11933 (52.6)	2355 (42.0)	16607 (43.8)	2473 (39.8)	1215 (34.7)	168 (21.1)
Female	226183 (55.4)	11067 (45.3)	434 (41.0)	13997 (43.6)	1044 (52.1)	13472 (49.3)	10735 (47.4)	3257 (58.0)	21293 (56.2)	3745 (60.2)	2290 (65.3)	628 (78.9)
**Age (mean (SD))**	56.43 (8.06)	58.74 (7.91)	59.82 (7.26)	61.11 (7.06)	59.68 (7.79)	59.57 (7.83)	60.26 (7.53)	56.66 (8.35)	60.53 (7.37)	59.84 (7.60)	58.05 (7.99)	57.71 (8.85)
**BMI (mean (SD))**	27.16 (4.59)	29.84 (6.24)	30.72 (6.50)	28.49 (5.56)	28.51 (5.64)	28.97 (5.65)	29.15 (5.71)	28.36 (5.45)	28.55 (5.43)	28.10 (5.10)	28.60 (5.74)	30.01 (6.71)
**Waist-hip-ratio (mean (SD))**	0.87 (0.09)	0.91 (0.09)	0.92 (0.09)	0.91 (0.09)	0.90 (0.09)	0.90 (0.09)	0.91 (0.09)	0.88 (0.09)	0.89 (0.09)	0.88 (0.10)	0.88 (0.09)	0.88 (0.09)
**Diagnosed diabetes (%)**												
Yes	16602 (4.1)	3287 (13.5)	151 (14.3)	3835 (12.0)	244 (12.2)	3220 (11.8)	2884 (12.7)	461 (8.2)	4435 (11.7)	547 (8.8)	440 (12.6)	154 (19.3)
No	389636 (95.5)	20955 (85.8)	899 (84.9)	27986 (87.3)	1746 (87.1)	23872 (87.3)	19568 (86.3)	5104 (90.9)	33173 (87.5)	5636 (90.6)	3032 (86.5)	632 (79.4)
Unknown	1917 (0.4)	191 (0.7)	9 (0.8)	253 (0.7)	15 (0.7)	254 (0.9)	216 (1.0)	47 (0.9)	292 (0.8)	35 (0.6)	33 (0.9)	10 (1.3)
**Townsend deprivation index (mean (SD))**	-1.40 (3.03)	-0.67 (3.35)	-0.59 (3.30)	-0.63 (3.37)	-0.48 (3.46)	-0.58 (3.39)	-0.53 (3.40)	-0.72 (3.34)	-0.87 (3.30)	-1.00 (3.26)	-0.65 (3.33)	-0.53 (3.35)
**Smoking status (%)**												
Current	39330 (9.6)	3613 (14.8)	155 (14.6)	5934 (18.5)	363 (18.1)	4058 (14.8)	3491 (15.4)	699 (12.5)	4644 (12.3)	703 (11.3)	463 (13.2)	123 (15.5)
Previous	136366 (33.4)	9308 (38.1)	387 (36.5)	13303 (41.5)	737 (36.8)	10914 (39.9)	9205 (40.6)	2071 (36.9)	14860 (39.2)	2482 (39.9)	1307 (37.3)	295 (37.1)

Never	230336 (56.4)	11290 (46.2)	504 (47.6)	12533 (39.1)	888 (44.3)	12085 (44.2)	9716 (42.9)	2800 (49.9)	18030 (47.6)	2993 (48.1)	1689 (48.2)	367 (46.1)
Unknown smoking	2123 (0.5)	222 (0.9)	13 (1.2)	304 (0.9)	17 (0.8)	289 (1.1)	256 (1.1)	42 (0.7)	366 (1.0)	40 (0.6)	46 (1.3)	11 (1.4)

Supplementary figure 2. Two sample Mendelian randomisation results for BMI and FinnGen infection outcomes. BMI SNP effect sizes shown against effect sizes for A) bacterial pneumonia, B) influenza, C) lower respiratory tract infections, D) upper respiratory tract infections, E) cystitis, and F) pyelonephritis. IVW, MR-Egger, median IV, and penalised median IV tests used.

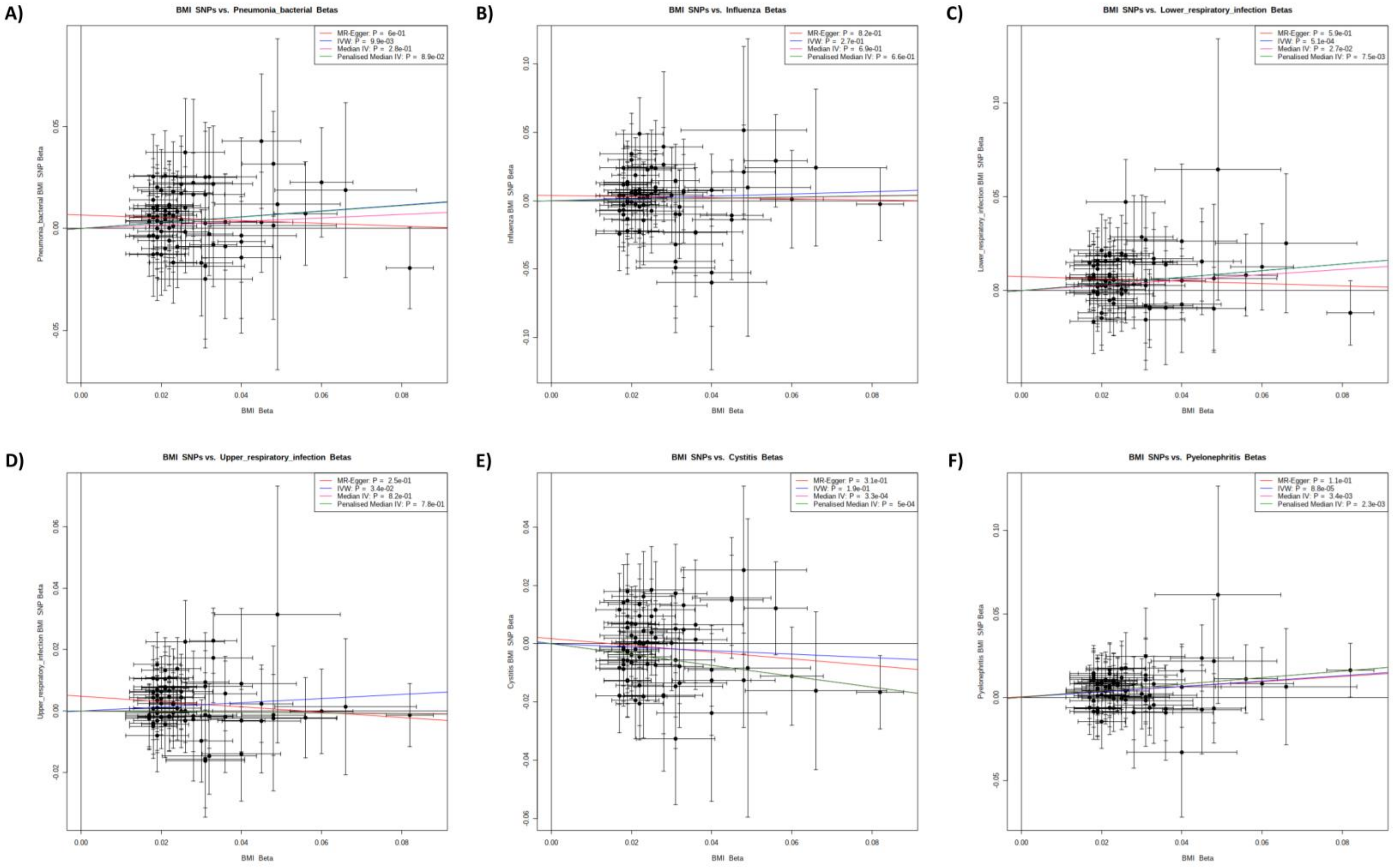

Supplementary table 3. MR PRESSO sensitivity analysis results for pleiotropy and correction for outliers in the association of BMI and infections.

Outcome	N SNPs	N outliers	Beta	SE	P value	Corrected Beta	Corrected SE	Corrected P value	Global P value	Distortion P value
Skin infection (bacterial)	71	0	0.404	0.03	<0.001	NA	NA	NA	0.761	NA
Dermatophytosis (fungal skin infection)	71	0	0.460	0.098	<0.001	NA	NA	NA	0.406	NA
Bacterial pneumonia	71	1	0.143	0.054	0.01	0.210	0.055	<0.001	0.046	0.31
Influenza	71	1	0.083	0.074	0.268	0.059	0.070	0.401	0.032	0.551
Lower respiratory tract infection	71	2	0.177	0.049	<0.001	0.218	0.047	<0.001	0.014	0.38
Upper respiratory tract infection	71	1	0.068	0.031	0.034	0.053	0.030	0.077	0.002	0.498
Cystitis	71	0	-0.060	0.046	0.194	NA	NA	NA	<1e-04	NA
Pyelonephritis	71	0	0.162	0.036	<0.001	NA	NA	NA	0.809	NA

Supplementary table 4. Observational associations of BMI and infections in primary and infection hospitalisation.

Infection	Primary care			Hospitalisation		
	N	Odds ratio (95%CI) per 5 kg/m^2	p value	N	Odds ratio (95%CI) per 5 kg/m^2	p value
Bacterial skin infection	49866	1.30 (1.29-1.32)	<0.001	24029	1.63 (1.61-1.65)	<0.001
Fungal skin infection	27553	1.19 (1.18-1.21)	<0.001	1033	1.87 (1.78-1.96)	<0.001
Bacterial pneumonia	5656	1.17 (1.13-1.20)	<0.001	31607	1.30 (1.29-1.32)	<0.001
Influenza	13492	1.20 (1.18-2.22)	<0.001	1954	1.32 (1.26-1.38)	<0.001
Non-influenza viral respiratory tract infection	112212	1.21 (1.20-1.23)	<0.001	26940	1.43 (1.41-1.45)	<0.001
Lower respiratory tract infection	75587	1.28 (1.27-1.30)	<0.001	22306	1.48 (1.46-1.50)	<0.001
Upper respiratory tract infection	69201	1.21 (1.20-1.22)	<0.001	5545	1.28 (1.25-1.32)	<0.001
Bacterial urinary tract infection	42751	1.13 (1.11-1.14)	<0.001	37302	1.32 (1.31-1.34)	<0.001
Cystitis	41657	1.13 (1.11-1.14)	<0.001	6149	1.22 (1.19-1.25)	<0.001
Pyelonephritis	2136	1.14 (1.09-1.19)	<0.001	3436	1.34 (1.30-1.38)	<0.001
Fungal genital infection	17180	1.13 (1.11-1.15)	<0.001	769	1.63 (1.54-1.72)	<0.001

Supplementary table 5. One sample Mendelian randomisation associations of BMI on infections in primary and infection hospitalisation.

Infection	Primary care			Hospitalisation		
	N	Odds ratio (95%CI) per 5 kg/m^2	p value	N	Odds ratio (95%CI) per 5 kg/m^2	p value
Bacterial skin infection	39078	1.37 (1.24-1.53)	<0.001	18540	1.93 (1.71-2.19)	<0.001
Fungal skin infection	21449	1.34 (1.18-1.53)	<0.001	806	2.81 (1.58-4.97)	<0.001
Bacterial pneumonia	4513	1.06 (0.83-1.36)	0.641	24438	1.42 (1.27-1.58)	<0.001
Influenza	10304	1.18 (0.99-1.40)	0.062	1486	1.14 (0.75-1.74)	0.538
Non-influenza viral respiratory tract infection	87346	1.15 (1.06-1.26)	0.001	20293	1.39 (1.24-1.57)	<0.001
Lower respiratory tract infection	58999	1.22 (1.11-1.34)	<0.001	16839	1.43 (1.25-1.62)	<0.001
Upper respiratory tract infection	53257	1.12 (1.01-1.23)	0.027	4107	1.37 (1.06-1.77)	0.015
Bacterial urinary tract infection	33553	1.06 (0.94-1.18)	0.359	28600	1.19 (1.07-1.32)	<0.001
Cystitis	32696	1.06 (0.94-1.19)	0.329	4735	0.77 (0.61-0.98)	0.032
Pyelonephritis	1659	1.03 (0.69-1.55)	0.874	2543	1.18 (0.85-1.62)	0.326
Fungal genital infection	13409	1.12 (0.96-1.31)	0.156	592	1.55 (0.80-3.02)	0.196

Supplementary table 6. Two sample Mendelian randomisation results for BMI on infections. IVW, MR-Egger, median IV, and penalised median IV tests used.

Trait	betaIVW2	sebetaIVW2	tIVW	pIVW	IVR_int	p.hetero	betaEgger	sebetaEgger	tegger	pEgger	egger_int	int_p	betaWM	sebetaWM	tWM	pWM	betaPWM	sebetaPWM	tPWM	pPWM	n_snp
Skin infection (bacterial)	0.404	0.041	9.980	0.000	0	0.722	0.396	0.099	3.991	0.000	0.000	0.918	0.382	0.066	5.754	0.000	0.380	0.064	5.972	0.000	71
Dermatophytosis (fungal skin infection)	0.460	0.098	4.713	0.000	0	0.394	0.483	0.240	2.009	0.048	-0.001	0.916	0.475	0.152	3.117	0.002	0.472	0.156	3.031	0.002	71
Bacterial pneumonia	0.143	0.054	2.650	0.010	0	0.065	-0.068	0.130	0.522	0.604	0.007	0.080	0.086	0.080	1.071	0.284	0.140	0.082	1.698	0.089	71
Influenza	0.083	0.074	1.117	0.268	0	0.030	-0.042	0.182	0.230	0.819	0.004	0.455	0.041	0.103	0.401	0.688	0.046	0.106	0.436	0.663	71
Lower respiratory tract infection	0.177	0.049	3.643	0.001	0	0.018	-0.062	0.116	0.536	0.594	0.007	0.027	0.141	0.064	2.208	0.027	0.177	0.066	2.676	0.007	71
Upper respiratory tract infection	0.068	0.031	2.168	0.034	0	0.001	-0.086	0.074	1.164	0.248	0.005	0.026	-0.009	0.042	0.229	0.819	-0.012	0.041	0.283	0.777	71
Cystitis	-0.060	0.046	1.311	0.194	0	0.000	-0.116	0.112	1.029	0.307	0.002	0.588	-0.186	0.052	3.593	0.000	-0.186	0.054	3.482	0.000	71
Pyelonephritis	0.162	0.039	4.164	0.000	0	0.794	0.152	0.095	1.597	0.115	0.000	0.902	0.196	0.067	2.932	0.003	0.196	0.064	3.047	0.002	71

Supplementary table 7. Observational associations of BMI and infections in primary and infection hospitalisation, stratified by individuals with/without diabetes.

Infection	Outcome source	Diabetes		No diabetes	
		N	Odds ratio (95%CI) per 5 kg/m^2	N	Odds ratio (95%CI) per 5 kg/m^2
Bacterial skin infection	Hospitalisation	3208	1.50 (1.45-1.55)	20667	1.55 (1.53-1.57)
Bacterial skin infection	Primary care	1489	1.62 (1.53-1.71)	9676	1.62 (1.59-1.65)
Fungal skin infection	Hospitalisation	145	1.83 (1.63-2.05)	881	1.73 (1.63-1.83)
Fungal skin infection	Primary care	1784	1.18 (1.12-1.24)	25650	1.18 (1.16-1.2)
Bacterial pneumonia	Hospitalisation	3752	1.29 (1.25-1.33)	27648	1.24 (1.22-1.26)
Bacterial pneumonia	Primary care	478	1.22 (1.12-1.33)	5156	1.12 (1.09-1.16)
Influenza	Hospitalisation	235	1.27 (1.14-1.41)	1707	1.24 (1.18-1.3)
Influenza	Primary care	801	1.12 (1.05-1.20)	12626	1.18 (1.16-1.20)
Non-influenza viral respiratory tract infection	Hospitalisation	3149	1.31 (1.26-1.35)	23591	1.38 (1.36-1.40)
Non-influenza viral respiratory tract infection	Primary care	6798	1.20 (1.15-1.24)	104936	1.18 (1.17-1.19)
Lower respiratory tract infection	Hospitalisation	2817	1.32 (1.28-1.37)	19318	1.42 (1.40-1.44)
Lower respiratory tract infection	Primary care	5167	1.25 (1.20-1.30)	70094	1.25 (1.24-1.27)
Upper respiratory tract infection	Hospitalisation	454	1.26 (1.17-1.36)	5054	1.24 (1.21-1.28)
Upper respiratory tract infection	Primary care	4041	1.18 (1.13-1.23)	64849	1.17 (1.16-1.19)
Bacterial urinary tract infection	Hospitalisation	4339	1.28 (1.24-1.32)	32729	1.26 (1.25-1.28)
Bacterial urinary tract infection	Primary care	2557	1.23 (1.18-1.29)	40017	1.05 (1.03-1.06)
Cystitis	Hospitalisation	539	1.22 (1.14-1.31)	5580	1.18 (1.15-1.21)
Cystitis	Primary care	2460	1.23 (1.18-1.29)	39026	1.05 (1.03-1.06)

Pyelonephritis	Hospitalisation	427	1.26 (1.17-1.36)	2980	1.27 (1.22-1.31)
Pyelonephritis	Primary care	179	1.23 (1.09-1.39)	1945	1.04 (0.99-1.09)
Fungal genital infection	Hospitalisation	145	1.52 (1.34-1.72)	616	1.5 (1.39-1.60)
Fungal genital infection	Primary care	1228	1.28 (1.21-1.35)	15888	1.02 (1.00-1.04)

Supplementary table 8. One sample Mendelian randomisation associations of BMI on infections in primary and infection hospitalisation, stratified by individuals with/without diabetes.

Infection	Outcome source	Diabetes		No diabetes	
		N	Odds ratio (95%CI) per 5 kg/m^2	N	Odds ratio (95%CI) per 5 kg/m^2
Bacterial skin infection	Hospitalisation	2324	1.64 (1.25-2.14)	16146	1.83 (1.58-2.10)
Bacterial skin infection	Primary care	1094	1.76 (1.13-2.75)	7658	2.06 (1.66-2.56)
Fungal skin infection	Hospitalisation	102	4.06 (1.27-13.00)	700	2.39 (1.23-4.62)
Fungal skin infection	Primary care	1265	1.54 (1.03-2.32)	20133	1.31 (1.14-1.51)
Bacterial pneumonia	Hospitalisation	2691	1.28 (0.99-1.65)	21645	1.33 (1.17-1.51)
Bacterial pneumonia	Primary care	359	1.18 (0.61-2.26)	4146	1.00 (0.76-1.33)
Influenza	Hospitalisation	171	1.05 (0.43-2.55)	1312	1.04 (0.64-1.69)
Influenza	Primary care	538	1.62 (0.93-2.81)	9742	1.12 (0.93-1.36)
Non-influenza viral respiratory tract infection	Hospitalisation	2164	1.31 (0.99-1.72)	18045	1.31 (1.14-1.50)
Non-influenza viral respiratory tract infection	Primary care	4909	1.23 (0.92-1.65)	82242	1.12 (1.02-1.23)
Lower respiratory tract infection	Hospitalisation	1948	1.28 (0.96-1.71)	14819	1.34 (1.15-1.55)
Lower respiratory tract infection	Primary care	3785	1.36 (1.00-1.84)	55067	1.17 (1.05-1.30)
Upper respiratory tract infection	Hospitalisation	292	1.53 (0.77-3.03)	3797	1.32 (0.99-1.75)
Upper respiratory tract infection	Primary care	2817	1.30 (0.94-1.80)	50324	1.07 (0.97-1.19)
Bacterial urinary tract infection	Hospitalisation	3086	1.28 (1.01-1.63)	25412	1.07 (0.96-1.21)
Bacterial urinary tract infection	Primary care	1866	1.29 (0.9-1.85)	31610	0.98 (0.87-1.10)
Cystitis	Hospitalisation	392	0.81 (0.45-1.48)	4326	0.71 (0.55-0.93)
Cystitis	Primary care	1788	1.29 (0.89-1.85)	30833	0.98 (0.87-1.11)

Pyelonephritis	Hospitalisation	295	1.79 (0.90-3.54)	2232	0.97 (0.67-1.40)
Pyelonephritis	Primary care	145	1.22 (0.46-3.25)	1506	0.89 (0.57-1.41)
Fungal genital infection	Hospitalisation	106	0.83 (0.27-2.57)	482	1.6 (0.72-3.54)
Fungal genital infection	Primary care	906	1.32 (0.84-2.08)	12470	1.04 (0.87-1.23)
