# Supplementary material for: Higher Body Mass Index Is a Causal Risk Factor for Skin Infections: A Mendelian Randomisation Study Using UK Biobank and FinnGen
Source: Diabetes Obes Metab. 2026 Apr 20;28(7):5907–14. doi: 10.1111/dom.70797 (PMC13243997; doi:10.1111/dom.70797)
Supplement: Supplementary file 2 — Data S1. dom70797‐sup‐0002‐supinfo.pdf. [file DOM-28-5907-s001.pdf]

Appendix

STROBE-MR checklist of recommended items to address in reports of Mendelian randomization studies[1 2]

Item No.	Section	Checklist item	Page No.	Relevant text from manuscript
1	**TITLE and ABSTRACT**	Indicate Mendelian randomization (MR) as the study's design in the title and/or the abstract if that is a main purpose of the study	1,2	Title: "Higher body mass index is a causal risk factor for skin infections: a Mendelian randomisation study using UK Biobank and FinnGen"<br><br>Abstract: "We used Mendelian randomisation to evaluate the potential causal role of higher BMI on common bacterial, viral, and fungal infections."
	**INTRODUCTION**			
2	**Background**	Explain the scientific background and rationale for the reported study. What is the exposure? Is a potential causal relationship between exposure and outcome plausible? Justify why MR is a helpful method to address the study question	3	"Higher BMI has been found to be associated with an increased risk of a range of common infections, including skin infections, respiratory infections, sepsis, and urinary tract infections. (7-10) This was further highlighted during the Covid-19 pandemic when increased BMI was found to be associated with poor infection outcomes such as hospitalisation. (11-13) However, risk factor associations reported for BMI have been almost entirely observational. There is limited evidence evaluating the causality of this association

				and so the potential causal role of BMI in infections is uncertain.”<br><br>“Causal inference methods such as Mendelian randomisation (MR), can be used to test for causal relationships using observational data. MR is an epidemiological method that uses genetic variation as an unconfounded proxy for the exposure (a person’s genotype is randomly assigned at birth) to test for a causal relationship with an outcome. (16, 17) This method is becoming increasingly popular to overcome unmeasured confounding and reverse causality, major limitations in observational studies. (16-19)”
3	**Objectives**	State specific objectives clearly, including pre-specified causal hypotheses (if any). State that MR is a method that, under specific assumptions, intends to estimate causal effects	4	“We therefore aimed to use an MR approach to test for a causal effect of higher BMI on common infections using large-scale population-based data.”<br><br>“Causal inference methods such as Mendelian randomisation (MR), can be used to test for causal relationships using observational data.”
	**METHODS**			
4	**Study design and data sources**	Present key elements of the study design early in the article. Consider including a table listing sources of data for all phases of the study. For each data source contributing to the analysis, describe the following:		

	a)	Setting: Describe the study design and the underlying population, if possible. Describe the setting, locations, and relevant dates, including periods of recruitment, exposure, follow-up, and data collection, when available.	4	“We used data from the UK Biobank and FinnGen, two of the largest datasets in the world combining genetic data and health information.<br><br>The UK Biobank is a large biomedical database containing health information and array-based genotyping data on around 500,000 participants. (21) The UK Biobank participants were volunteers recruited aged 40-70 between 2006 and 2010 across the UK. The baseline data are linked to electronic health record data for hospital admissions, and in a subset (n=230,542), primary care records.<br><br>FinnGen is a large-scale genomics initiative that has analysed over 500,000 Finnish biobank samples and correlated genetic variation with health data to understand disease mechanisms and predispositions. (22)”
	b)	Participants: Give the eligibility criteria, and the sources and methods of selection of participants. Report the sample size, and whether any power or sample size calculations were carried out prior to the main analysis	4	“The UK Biobank is a large biomedical database containing health information and array-based genotyping data on around 500,000 participants. (21) The UK Biobank participants were volunteers recruited aged 40-70 between 2006 and 2010 across the UK. The baseline data are linked to electronic health record data for hospital admissions, and in a subset (n=230,542), primary care records.

				FinnGen is a large-scale genomics initiative that has analysed over 500,000 Finnish biobank samples and correlated genetic variation with health data to understand disease mechanisms and predispositions. (22)”
	c)	Describe measurement, quality control and selection of genetic variants	5-6	“The genetic variants used for BMI were obtained from a 2015 genome-wide association study (GWAS) of 339 224 individuals that reported 97 genome-wide significant loci. (23) We excluded sex-specific variants and those with potential pleiotropy or secondary signals within a locus, resulting in 72 variants used in our analysis. The study used to derive genetic instruments did not use the UK Biobank, which minimises the overlap of samples used for genotype-exposure and genotype-outcome associations. Supplementary table 1 lists the genetic variants used.”
	d)	For each exposure, outcome, and other relevant variables, describe methods of assessment and diagnostic criteria for diseases	4-5	“Using UK Biobank linked electronic health record datasets, for each of these infection types we defined two outcomes: infection treated in primary care, and hospitalisation with infection. This allowed us to evaluate the potential impact of BMI on both mild and severe infection outcomes. We defined primary care infections as any Read code for the infection type in the primary care dataset. We defined hospitalisation as an International Classification of Diseases 10th Revision (ICD-10) code for the infection

type as any diagnosis in the Hospital Episodes Statistics (HES) dataset. The Read and ICD-10 codes used to define each infection type are available in a Github repository: https://github.com/rhianhopkins/UKBiobank-MRInfections. Individuals without a diagnosis any of the studied infection types were used as controls in the respective analyses.

In FinnGen, we selected the closest matching outcomes to the ones we defined above in the UK Biobank. These were: L12_INFECT_SKIN (bacterial skin infections), AB1_DERMATOPHYTOSIS (fungal skin infection), J10_PNEUMOBACT (bacterial pneumonia), J10_INFLUENZA (influenza), J10_LOWERINF (lower respiratory tract infections), J10_UPPERINFEC (upper respiratory tract infections), N14_CYSTITIS (cystitis), and N14_PYELONEPHR (pyelonephritis). There was no closely matching outcome in FinnGen for fungal genital infections."

"We defined BMI using the values recorded in the UK Biobank baseline assessment data. Individuals with missing values for BMI were excluded from the analyses (n=1,409)."

		e)	Provide details of ethics committee approval and participant informed consent, if relevant	14	“Ethics approval for the UK Biobank study was obtained from the North West Centre for Research Ethics Committee (11/NW/0382). (21) Written informed consent was obtained from all participants.”
5	**Assumptions**		Explicitly state the three core IV assumptions for the main analysis (relevance, independence and exclusion restriction) as well assumptions for any additional or sensitivity analysis	6	“Mendelian randomisation methods rely on three core assumptions: (24)<br>1. Relevance: the genetic instrument is associated with the exposure<br>2. Independence: the genetic instrument is not associated with confounders<br>3. Exclusion restriction: the genetic instrument influences the outcome only through the exposure<br>Additionally, an important assumption is gene-environment equivalence (the genetic instrument influences an environmental exposure equivalently to a proposed intervention that changes the population distribution of the exposure). (25)”
6	**Statistical methods: main analysis**		Describe statistical methods and statistics used		
		a)	Describe how quantitative variables were handled in the analyses (i.e., scale, units, model)	6	“We tested for observational associations between BMI and infection outcomes using a logistic regression model adjusted for age and sex in the UK Biobank cohort. Effect

					sizes were reported as odds ratios per 5 kg/m2 BMI increase.”
		b)	Describe how genetic variants were handled in the analyses and, if applicable, how their weights were selected	6	“We first combined genetic variants for BMI into a genetic risk score (GRS) using the published effect size for each SNP as weights.”
		c)	Describe the MR estimator (e.g. two-stage least squares, Wald ratio) and related statistics. Detail the included covariates and, in case of two-sample MR, whether the same covariate set was used for adjustment in the two samples	6-7	“In the first stage of the two-stage least squares approach, the normalised GRS was regressed against BMI using a linear regression to derive genetically predicted exposure values. In the second stage, the genetically predicted exposure was regressed against the infection outcome in a logistic regression model, adjusted for age, sex, and the residuals.”
		d)	Explain how missing data were addressed	5	“Individuals with missing values for BMI were excluded from the analyses (n=1,409).”<br>“We excluded individuals from the subsequent analyses if we could not calculate a genetic risk score.”
		e)	If applicable, indicate how multiple testing was addressed		NA
7	**Assessment of assumptions**		Describe any methods or prior knowledge used to assess the assumptions or justify their validity	5,7	“performed sensitivity analyses using additional methods that are more robust to potential violations of the standard MR assumptions (MR-Egger, median IV, penalised median IV). MR Egger is robust to pleiotropy and assesses whether genetic variants have pleiotropic effects on the outcome that differ on average from zero

				(indicated by the intercept). (26, 27) Median IV uses the median of the causal estimates for each genetic variant and allows up to 50% to be invalid instruments, and penalised median IV allows more precise causal estimates to contribute more weight to the analysis. (27, 28)”<br>“The genetic variants used for BMI were obtained from a 2015 genome-wide association study (GWAS) of 339 224 individuals that reported 97 genome-wide significant loci. (23)”
8	**Sensitivity analyses and additional analyses**	Describe any sensitivity analyses or additional analyses performed (e.g. comparison of effect estimates from different approaches, independent replication, bias analytic techniques, validation of instruments, simulations)	7	“performed sensitivity analyses using additional methods that are more robust to potential violations of the standard MR assumptions (MR-Egger, median IV, penalised median IV). MR Egger is robust to pleiotropy and assesses whether genetic variants have pleiotropic effects on the outcome that differ on average from zero (indicated by the intercept). (26, 27) Median IV uses the median of the causal estimates for each genetic variant and allows up to 50% to be invalid instruments, and penalised median IV allows more precise causal estimates to contribute more weight to the analysis. (27, 28)”
9	**Software and pre-registration**			
	a)	Name statistical software and package(s), including version and settings used	7	“Statistical analysis was performed using R version 4.3.0,”

		b)	State whether the study protocol and details were pre-registered (as well as when and where)		NA
	**RESULTS**				
10	**Descriptive data**				
		a)	Report the numbers of individuals at each stage of included studies and reasons for exclusion. Consider use of a flow diagram		Supplementary figures 1A and 1B
		b)	Report summary statistics for phenotypic exposure(s), outcome(s), and other relevant variables (e.g. means, SDs, proportions)		Table 1
		c)	If the data sources include meta-analyses of previous studies, provide the assessments of heterogeneity across these studies		NA
		d)	For two-sample MR:<br>i. Provide justification of the similarity of the genetic variant-exposure associations between the exposure and outcome samples<br>ii. Provide information on the number of individuals who overlap between the exposure and outcome studies	7	“We obtained the effect sizes for the BMI genetic variants from the 2015 GWAS study described above (Supplementary table 1). To obtain genotype-outcome associations for these SNPs, we used publicly available summary statistics from FinnGen (22) for each of the infection outcomes.”
11	**Main results**				
		a)	Report the associations between genetic variant and exposure, and between genetic variant and outcome, preferably on an interpretable scale		Supplementary table 1 and Supplementary table 5
		b)	Report MR estimates of the relationship between exposure and outcome, and the measures of uncertainty from the MR analysis, on an interpretable scale, such as odds ratio or relative risk per SD difference	8-9	“A causal association with BMI was seen for skin infections both in primary care (bacterial skin infections: Odds Ratio [OR] 1.37 [95%CI: 1.24-1.53] per 5 kg/m2 increase in BMI, p<0.001, fungal skin infections: OR 1.34 [95%CI: 1.18-1.53,

					p<0.001, Figure 1B) and for hospitalisation with skin infections (bacterial skin infections: OR 1.93 [95%CI: 1.71-2.19] per 5 kg/m2 increase in BMI, p<0.001, fungal skin infections: OR 2.81 [95%CI: 1.58-4.97, p<0.001, Figure 1A)."
		c)	If relevant, consider translating estimates of relative risk into absolute risk for a meaningful time period		NA
		d)	Consider plots to visualize results (e.g. forest plot, scatterplot of associations between genetic variants and outcome versus between genetic variants and exposure)		Figure 1 and Figure 2
12	**Assessment of assumptions**				
		a)	Report the assessment of the validity of the assumptions	9	"MR-Egger sensitivity suggests no evidence of pleiotropy (Figure 2A and Figure 2B)."<br>"Two-sample Mendelian randomisation MR-Egger suggests evidence of horizontal pleiotropy in the association of higher BMI and respiratory infections (Supplementary figure 2)."
		b)	Report any additional statistics (e.g., assessments of heterogeneity across genetic variants, such as I^2, Q statistic or E-value)		NA
13	**Sensitivity analyses and additional analyses**				
		a)	Report any sensitivity analyses to assess the robustness of the main results to violations of the assumptions	9	"MR-Egger sensitivity suggests no evidence of pleiotropy (Figure 2A and Figure 2B)."

				“Two-sample Mendelian randomisation MR-Egger suggests evidence of horizontal pleiotropy in the association of higher BMI and respiratory infections (Supplementary figure 2).”
		b) Report results from other sensitivity analyses or additional analyses		NA
		c) Report any assessment of direction of causal relationship (e.g., bidirectional MR)		NA
		d) When relevant, report and compare with estimates from non-MR analyses		NA
		e) Consider additional plots to visualize results (e.g., leave-one-out analyses)		NA
	**DISCUSSION**			
14	**Key results**	Summarize key results with reference to study objectives	10	“Using MR in large-scale population-based data, we demonstrate strong evidence of a causal role of increased BMI on bacterial and fungal infections. This causal effect was seen for milder cases of skin infection in primary care and hospitalisation with skin infections, and evidence was consistent in observational analyses and one- and two-sample MR. The effect sizes identified are highly clinically relevant, with a doubling of risk for hospitalisation with skin infection for every 5kg/m2 increase in BMI. Sensitivity analyses suggest this causal effect is robust to pleiotropy.

				In observational and one-sample MR analyses we found evidence of a causal role of higher BMI on some respiratory infections. However, two-sample MR sensitivity analyses suggest that this association is affected by horizontal pleiotropy and the genetic variants may be influencing infections through a pathway outside of BMI. This therefore violates the core MR assumptions. We also found little evidence of a causal effect of BMI on urogenital infections.”
15	**Limitations**	Discuss limitations of the study, taking into account the validity of the IV assumptions, other sources of potential bias, and imprecision. Discuss both direction and magnitude of any potential bias and any efforts to address them	12	“There are potential limitations to using data from the UK Biobank as the study recruited only individuals between the ages of 40-69 and there is a bias towards healthy individuals. The majority of UK Biobank are individuals of European ancestry and the numbers of individuals of other ancestries are too small to perform separate subgroup analyses in these groups. The summary statistics used in our two-sample MR also came from studies of Europeans only, and so associations may not be generalisable to other ancestries.”<br><br>“We were unable to use two-sample MR to test for a causal association of BMI and fungal genital infections due to the lack of a close matching outcome in FinnGen, however one-sample MR suggested no evidence of a causal effect of higher BMI on this infection type. As with all studies involving health record data,

			misclassification of infection outcomes is possible and relies on correct coding in the records. In the UK hospital coding is performed by professional clinical coders and primary care coding usually performed by the clinician making the diagnosis. Using data from both sources mitigated against the impact of any systemic miscoding errors.”
16	**Interpretation**		
	a) Meaning: Give a cautious overall interpretation of results in the context of their limitations and in comparison with other studies	10	“Given the significant morbidity and mortality due to infections, the evidence we have found of a causal effect of BMI on bacterial and fungal skin infections provides a potential important target for intervention. Our study suggests it is likely that weight loss can reduce the risk of these infections. As we found a causal association with infections in primary care and in hospital, this suggests that higher BMI could both cause increased risk of getting an infection and it being severe enough to be hospitalised. This evidence aligns with a previous study finding higher BMI was causally associated with increased risk of hospitalisation for skin infection. (20) Our study builds on this by providing evidence of a causal effect of higher BMI on milder cases of skin infection treated in primary care, stratifying by bacterial and fungal infection aetiologies, and validating using two-sample MR in a different population.”

	b) Mechanism: Discuss underlying biological mechanisms that could drive a potential causal relationship between the investigated exposure and the outcome, and whether the gene-environment equivalence assumption is reasonable. Use causal language carefully, clarifying that IV estimates may provide causal effects only under certain assumptions	11	“There are several plausible mechanisms which may explain why higher BMI causing infections. Intertrigo is a skin condition where increased skin folds result in areas of increased friction and moisture retention, making them susceptible to bacterial and fungal infections. (30, 31) Additionally, changes in blood flow within the skin due to obesity may impair the immune response to infections and lead to skin barrier impairment. (31) Skin conditions such as lymphoedema and venous insufficiency that are caused by obesity can also cause localised skin barrier breaches that can lead to infection. (30, 31)”
	c) Clinical relevance: Discuss whether the results have clinical or public policy relevance, and to what extent they inform effect sizes of possible interventions	11	“The robust evidence we provide demonstrating a causal role of BMI in skin infections highlights that an emphasis on weight management is needed for skin infection prevention. While weight loss strategies need to be tailored to an individual, in the last few years there have been an increasing number of new medications that are available which can help weight loss, such as the next generation incretin-based agents. (32) Pharmacological intervention could therefore offer an effective intervention to support weight loss and improve infection outcomes. These weight loss interventions are expensive and therefore need to be targeted to those with the greatest benefit. Our findings suggest weight loss

				interventions could be particularly helpful to people who are most vulnerable to skin infections, for example people with diabetes (14) and people who have been admitted to hospital with a skin infection who are at high risk of being readmitted with another. (15)”
17	**Generalizability**	Discuss the generalizability of the study results (a) to other populations, (b) across other exposure periods/timings, and (c) across other levels of exposure	12	“The majority of UK Biobank are individuals of European ancestry and the numbers of individuals of other ancestries are too small to perform separate subgroup analyses in these groups. The summary statistics used in our two-sample MR also came from studies of Europeans only, and so associations may not be generalisable to other ancestries.”
	**OTHER INFORMATION**			
18	**Funding**	Describe sources of funding and the role of funders in the present study and, if applicable, sources of funding for the databases and original study or studies on which the present study is based	13	“JMD is supported by a Wellcome Trust Early Career award (227070/Z/23/Z). This study was supported by the National Institute for Health and Care Research Exeter Biomedical Research Centre. The views expressed are those of the author(s) and not necessarily those of the NIHR or the Department of Health and Social Care.”
19	**Data and data sharing**	Provide the data used to perform all analyses or report where and how the data can be accessed, and reference these sources in the article. Provide the statistical code needed to reproduce the results	14	“All individual-level data used in this paper were obtained from the UK Biobank resource, and can be obtained from the UK Biobank at https://www.ukbiobank.ac.uk/enable-your-

		in the article, or report whether the code is publicly accessible and if so, where		research/apply-for-access. Access to summary statistics from the FinnGen resource can be obtained at: https://www.finngen.fi/en/access_results/.”
20	**Conflicts of Interest**	All authors should declare all potential conflicts of interest	13	“APM received prior research funding from Eli Lilly and Company, Pfizer, and AstraZeneca outside of the submitted work. All other authors declare no other relationships or activities that could appear to have influenced the submitted work.”

This checklist is copyrighted by the Equator Network under the Creative Commons Attribution 3.0 Unported (CC BY 3.0) license.

1. Skrivankova VW, Richmond RC, Woolf BAR, Yarmolinsky J, Davies NM, Swanson SA, et al. Strengthening the Reporting of Observational Studies in Epidemiology using Mendelian Randomization (STROBE-MR) Statement. JAMA. 2021;under review.

2. Skrivankova VW, Richmond RC, Woolf BAR, Davies NM, Swanson SA, VanderWeele TJ, et al. Strengthening the Reporting of Observational Studies in Epidemiology using Mendelian Randomisation (STROBE-MR): Explanation and Elaboration. BMJ. 2021;375:n2233.
